# Supplementary material for: The causal role of circulating inflammatory markers in osteoporosis: a bidirectional Mendelian randomized study
Source: Front Immunol. 2024 Jul 18;15:1412298. doi: 10.3389/fimmu.2024.1412298 (PMC11291241; doi:10.3389/fimmu.2024.1412298)
Supplement: Supplementary file 1 [file DataSheet_1.zip › supplementary material/CIM-OSTEOPOROSIS /GCST90274830.csv_leave_one_out.pdf]

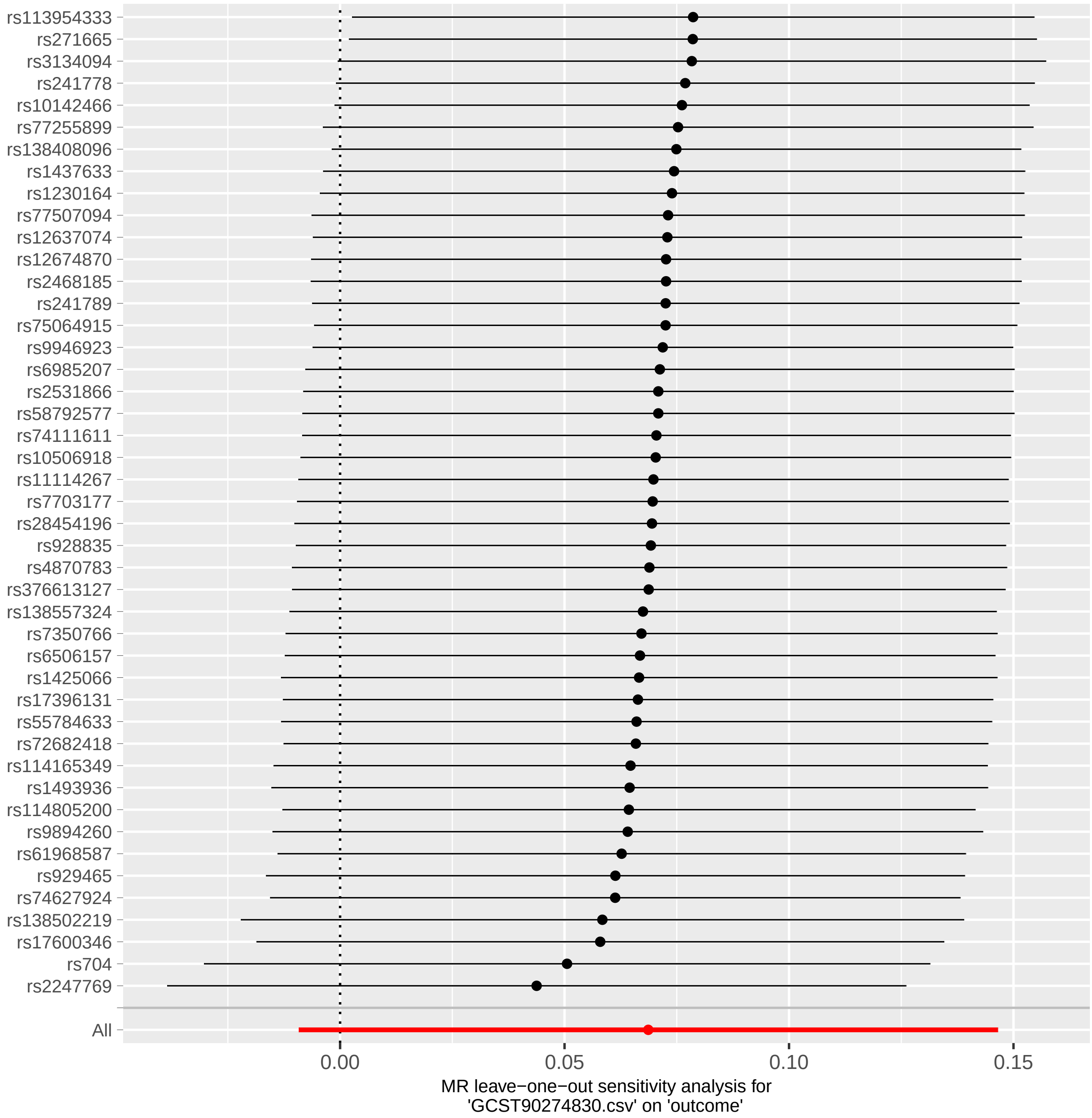
rs113954333
rs271665
rs3134094
rs241778
rs10142466
rs77255899
rs138408096
rs1437633
rs1230164
rs77507094
rs12637074
rs12674870
rs2468185
rs241789
rs75064915
rs9946923
rs6985207
rs2531866
rs58792577
rs74111611
rs10506918
rs11114267
rs7703177
rs28454196
rs928835
rs4870783
rs376613127
rs138557324
rs7350766
rs6506157
rs1425066
rs17396131
rs55784633
rs72682418
rs114165349
rs1493936
rs114805200
rs9894260
rs61968587
rs929465
rs74627924
rs138502219
rs17600346
rs704
rs2247769
All
0.00
0.05
0.10
0.15
MR leave−one−out sensitivity analysis for
'GCST90274830.csv' on 'outcome'
